# miR-144/451 cluster plays an oncogenic role in esophageal cancer by inhibiting cell invasion

**DOI:** 10.1186/s12935-018-0679-8

**Published:** 2018-11-15

**Authors:** Zhikui Gao, Peng Zhang, Ming Xie, Han Gao, Lihong Yin, Ran Liu

**Affiliations:** 1 Key Laboratory of Environmental Medicine Engineering, Ministry of Education, School of Public Health, Southeast University, Nanjing, 210009 China; 2 Huzhou Center for Disease Control and Prevention, Huzhou, 313000 China; 3 North China Petroleum Bureau General Hospital, Renqiu, 062552 China

**Keywords:** Esophageal cancer, miRNA cluster, miR-144-3p, miR-144-5p, miR-451a

## Abstract

**Background:**

miRNA clusters are widely expressed across species, accumulating evidence has illustrated that miRNA cluster functioned more efficiently than single miRNA in cancer oncogenesis. It is likely that miRNA clusters are more stable and reliable than individual miRNA to be biomarkers for diagnosis and therapy. We previously found low expression of miR-144/451 was closely related with the risk for esophageal cancer. Researches on miR-144/451 cluster were mostly focused on individual miRNA but not the whole cluster, the regulatory mechanism of miRNA cluster were largely unknown.

**Methods:**

In present study, we firstly analysed biological functions of individual miRNAs of miR-144/451 in ECa9706 transfected with miRNA mimics. We further analysed the biological function of the whole cluster in stable transgenic cell overexpressing miR-144/451. We then performed genome-wide mRNA microarray to detect differentially expressed gene profiles in stable transgenic cells.

**Results:**

Overexpression of miR-144-3p promoted early apoptosis of ECa9706 and inhibited cell migration, cell invasion and cell proliferation. miR-144-5p and miR-451a inhibited cell proliferation, at the same time, miR-451a inhibited cell migration. Overexpression of miR-144/451 leads to the arrest cell cycle from S to G2 and G2 to M,while the invasion ability was obviously inhibited. We further observed c-Myc, p-ERK were downregulated in cells overexpressing miR-144/451, while p53 was up-regulated. The downstream effectors of c-Myc, MMP9 and p-cdc2 were downregulated in miR-144/451 stable transgenic cell. miR-144/451 may or partly inhibited cell cycles and invasion of ECa9706 through inhibiting ERK/c-Myc signaling pathway.

**Conclusion:**

Collectively, we analysed the function of miR-144/451 cluster from individual to overall level. miR-144/451 cluster played proto oncogene role in esophageal cancer by inhibiting cell invasion.

**Electronic supplementary material:**

The online version of this article (10.1186/s12935-018-0679-8) contains supplementary material, which is available to authorized users.

## Background

Esophageal cancer is a common digestive system cancer with high incidence and mortality. According to statistics of WHO, esophageal cancer ranks as the fourth highest cause of cancer-related mortality in the world, there are 456,000 new cases and 400,000 deaths in 2012, more than half of the new cases occurs in China [1]. It is worth mentioning the incidence is still growing. Although endoscopic resection is effective for esophageal cancer patients at early stage, the overall 5-year survival rate is still no more than 20% [2, 3]. There are no effective treatment for advanced esophageal cancer, so the key point for raising the survival rate of esophageal cancer is detecting and treating at early stage [4]. However, no obvious clinical symptom for esophageal cancer can be observed, searching for sensitive and specific biomarkers for early esophageal cancer becomes especially important.

Growing evidence showed that miRNAs played important roles in post transcriptional regulation by binding to the 3′UTR region of mRNAs, directing the repression of protein expression [5]. Abnormal miRNA-mediated regulation can affect oncellular functions which were closely related to the occurrence of tumor, miRNAs seem to be important molecular markers in the diagnosis of tumor [6, 7]. Usually, miRNA genes were transcribed under the regulation of promoter and operon, in particular some were closely aligned on chromosome formed as a cluster. These clusters are suspected to transcribe together, though not exclusively, mediate synergistic or antagonistic regulatory effects. miRNA cluster is likely to be more stable and reliable than individual miRNA to be biomarkers for diagnosis [8, 9]. However, most of the studies on miRNA clusters focused on individual miRNAs but not the cluster, regulatory mechanisms of miRNA cluster were largely unknown.

miR-144/451 cluster is highly conversed in different species, miRbase database (http://www.mirbase.org/) shows miR-144/451 cluster is constituted by miR-144-3p, miR-144-5p, miR-451a, miR-4732-3p and miR-4732-5p (Table 1). In previous study, we discovered the low-expression of miR-144/451 was closely related with the risk for esophageal cancer [10]. miR-144-3p was reported to be related with gastric carcinoma, lung cancer, hepatoma and colorectal cancer [11–14], miR-144-5p was reported to be related with bladder cancer and colorectal cancer [15, 16], miR-451a was reported to be related with lung cancer, gastric carcinoma, colorectal cancer, liver cancer, breast cancer and osteosarcoma [17–22]. However, few is known about the function and mechanism of miR-144/451 in esophageal cancer. Most of the researches about miR-144/451 is focused only on single miRNAs but not on the cluster.

**Table 1:** Location and sequences of miR-144/451 cluster

miRNA	Sequence	Location (*GRCh38*)
miR-144-3p	UACAGUAUAGAUGAUGUACU	chr17: 28861584–28861603
miR-144-5p	GGAUAUCAUCAUAUACUGUAAG	chr17: 28861547–28861568
miR-451a	AAACCGUUACCAUUACUGAGUU	chr17: 28861385–28861406
miR-4732-3p	GCCCUGACCUGUCCUGUUCUG	chr17: 28861663–28861685
miR-4732-5p	UGUAGAGCAGGGAGCAGGAAGCU	chr17: 28861697–28861717

In present study, we analysed functions of individual miRNA of miR-144/451 in ECa9706, we established stable transgenic cells overexpressing miR-144/451 and analysed the function of the cluster. To better understand the potential mechanism of the cluster, genome-wide mRNA microarray and Western blot were performed. Results of the present study may advance our understanding of the expression pattern and functional role of the miR-144/451 cluster in esophageal carcinoma.

## Materials and methods

### Cell line

ECa9706, ECa109, H5E46, Het-1A and HEK-293T cells were provided by Key Laboratory of Environmental Medicine Engineering, Ministry of Education, School of Public Health, Southeast University. Cells were grown in RPMI-1640 containing 10% fetal bovine serum (Gibico), 100 U/mL penicillin–streptomycin solution (Gibico) at 37 °C in incubator containing 5% CO_2_ humidified atmosphere. Transient transfected Cells over-expressing miRNAs were obtained using micrON™ miRNA mimic (RiboBio). Lipofectamine^®^ RNAiMAX Reagent (Invitrogen) were used for transfection according to manufacturer’s instructions. Stable expression cell strain over-expressing miR-144/451 cluster were obtained using lentiviral transfection.

### Plasmids construction, virus production, and infection of target cells

To obtain pri-miR-144/451 cluster plasmid, a synthetic sequence within the range of upstream 200 bp to downstream 200 bp of pri-miR-144/451 was cloned into PCDH-CMV-MCS-EF1-Puro lentivirus plasmid. Plasmids of pMDLg/pRRE, pRSV-Rev, pMD2G and PCDH-CMV-MCS-EF1-Puro were transfected into HEK-293T for packaging lentivirus.

### Antibodies and reagents

PTEN (138G6) Rabbit Monoclonal antibody, Phospho-cdc2 (Tyr15) antibody, Phospho-p44/42 MAPK (ERK1/2) (Thr202/Tyr204) Rabbit Monoclonal antibody, p44/42 MAPK (Erk1/2) Rabbit Monoclonal antibody, Phospho-β-Catenin (Ser33/37/Thr41) antibody, Total-β-Catenin antibody, Phospho-c-Myc (Ser62) Rabbit Monoclonal antibody, p53 Rabbit Monoclonal antibody, Non-phospho (Active) β-Catenin (Ser33/37/Thr41) Rabbit Monoclonal antibody were obtained from Cell Signaling Technology (CST). Mouse anti-human C-myc monoclonal antibody, anti-MMP-9 antibody were obtained from Millipore.

### Apoptosis and cell cycle

For cells transfected with mimics, cell apoptosis were detected using Annexin V-APC/7-AAD Apoptosis Detection Kit (KGA1026, KeyGEN BioTECH) according to the manufacture’s protocol, for cells stably transfected with Lentiviral vectors, cell apoptosis was detected using (KGA1026, KeyGEN BioTECH). Flow cytometry (PI staining) were used for detecting cell cycle using PI cell cycle Detection Kit (KGA107, KeyGEN BioTECH) according to the manufacture’s protocol, experiments were done in triplicates.

### Cell proliferation assay

Cell proliferation were detected using 5-ethynyl-2′-deoxyuridine (EdU) labeling/detection kit (Ribobio). Firstly, cells with a density of 1 × 104 cells/well were planted on 96-well plates, after 24 h, 50 mM EdU were added into the plate for an additional 2 h. Then the cells were fixed with 4% formaldehyde in PBS for 30 min and incubate with glycine for 5 min. After washing with PBS and 0.5% TritonX-100 in PBS, cells were incubated with 1 × Apollo dye at room temperature in dankness for 30 min. At last, wash cells with 0.5% TritonX-100 in PBS and methanol, incubate cells with 1 × Hoechst 33342 dye at room temperature in dankness for 30 min. Preserve labeled cells in PBS. Observe and photograph using fluorescence microscopy, select five images randomly for cell counting, Assays were performed with five parallels.

### Invasion and migration

Cell invasion and migration were detected using 8.0 μm Transwell chamber (Corning). For cell migration, 5 × 10^4^ cells were seeded into the upper chamber in serum-free media, the lower chamber were filled with RPMI-1640 containing 10% fetal bovine serum, culture for 24 h, color and count migrated cell. For cell invasion, 5 × 10^5^ cells were seeded into the upper chamber using serum-free media, the lower chamber were filled with RPMI-1640 containing 50% fetal bovine serum, culture for 24 h, color and count invasive cells, experiments were done in triplicates.

### RNA extraction and genome-wide mRNA microarray

Total RNA were isolated using Trizol reagent (Invitrogen). RNA concentration were measured using Nanodrop 2000 (Thermo Fisher) and Agilent 2100 Bioanalyzer (Agilent). aRNA were obtained using GeneChip 3′IVT PLUS Kit (Affymetrix). mRNA profiles were detected using Affymetrix GeneChip primeview human Expression Array (100 format) in miR-144/451 over-expressing and negative control cells, experiments were done in triplicates.

### Bioinformatics analysis of microarray and target prediction

Ingenuity Pathway Analysis (IPA) were performed for analysing mRNA profiles detected by genome-wide mRNA microarray.

### Western blot analysis

Cellular protein were extracted with cold RIPA buffer (Beyotime) containing protease and phosphatase inhibitors (Millipore). Lysates were cleared by centrifugation at 14,000 rpm at 4 °C for 15 min. Protein concentration were detected using bicinchoninic acid (BCA) assay (Thermo Fisher). Aliquots of protein (20 μg) were separated by 10% SDS-PAGE and the separated proteins were transferred onto 0.45 μm PVDF membrane (Millipore). Membranes were blocked with 5% (w/v) non-fat milk in Tris–HCl buffered saline (pH 7.4) with Tween-20 and incubated with the primary antibody overnight at 4 °C. Subsequently, membranes were washed with Tris–HCl buffered saline and incubated with secondary antibody conjugated to horseradish peroxidase respectively diluted in 1:1000, at room temperature for 1 h. Membranes were washed in Tris–HCl buffered saline and bounds were detected with SuperSignal West Femto/Pico Kit (Thermo Fisher). Blots were visualized and quantified using Tanon-5200 Imaging System (Tanon).

### RT-QPCR

All primers (Bulge-Loop™ miRNA RT-QPCR Primer kits) for miRNA were purchased from Guangzhou RiboBio Co., Ltd. All primers for mRNA were synthetized in GenScript Corporation, U6 was selected as internal reference for miRNAs. The Sequence for forward primer of pri-miRNA was ACAGTGCTTTTCAAGCCATGC, for reverse primer, the sequence was GGGTGCCCGGACTAGTACAT. β-actin was selected as internal reference for detecting pri-miRNA, sequence for forward primer was: ATCCGCAAAGACCTGT, and for reverse primer the sequence was: GGGTGTAACGCAACTAAG.

Total RNA (~ 2 μg) were extracted using Trizol regent (Invitrogen). cDNA was synthesized using Moloney Murine Leukemia Virus (MMLV) reverse transcriptase (Promega) and ribonuclease inhibitor (Thermo Fisher). SYBR Green mastermix were purchased from Toyobo Technologies. QPCR reactions were performed using StepOnePlus system (Applied Biosystems). Data for miRNA and mRNA were normalized using U6 and β-actin respectively. Expression of miRNA and mRNA were presented as relative RNA expression using ΔΔC_T_ formula (the fold change in target gene expression was equal to 2^−ΔΔCT^). All results were presented as mean of triplicates ± SD of three independent experiments.

### Statistical analysis

Statistical analysis was performed using SPSS 17.0. Group differences were explored by Student’s t-test and analysis of variance (ANOVA). *P* value < 0.05 was considered to be statistically significant.

## Results

### Expression of miR-144/451 in different cells

**Fig. 1:**
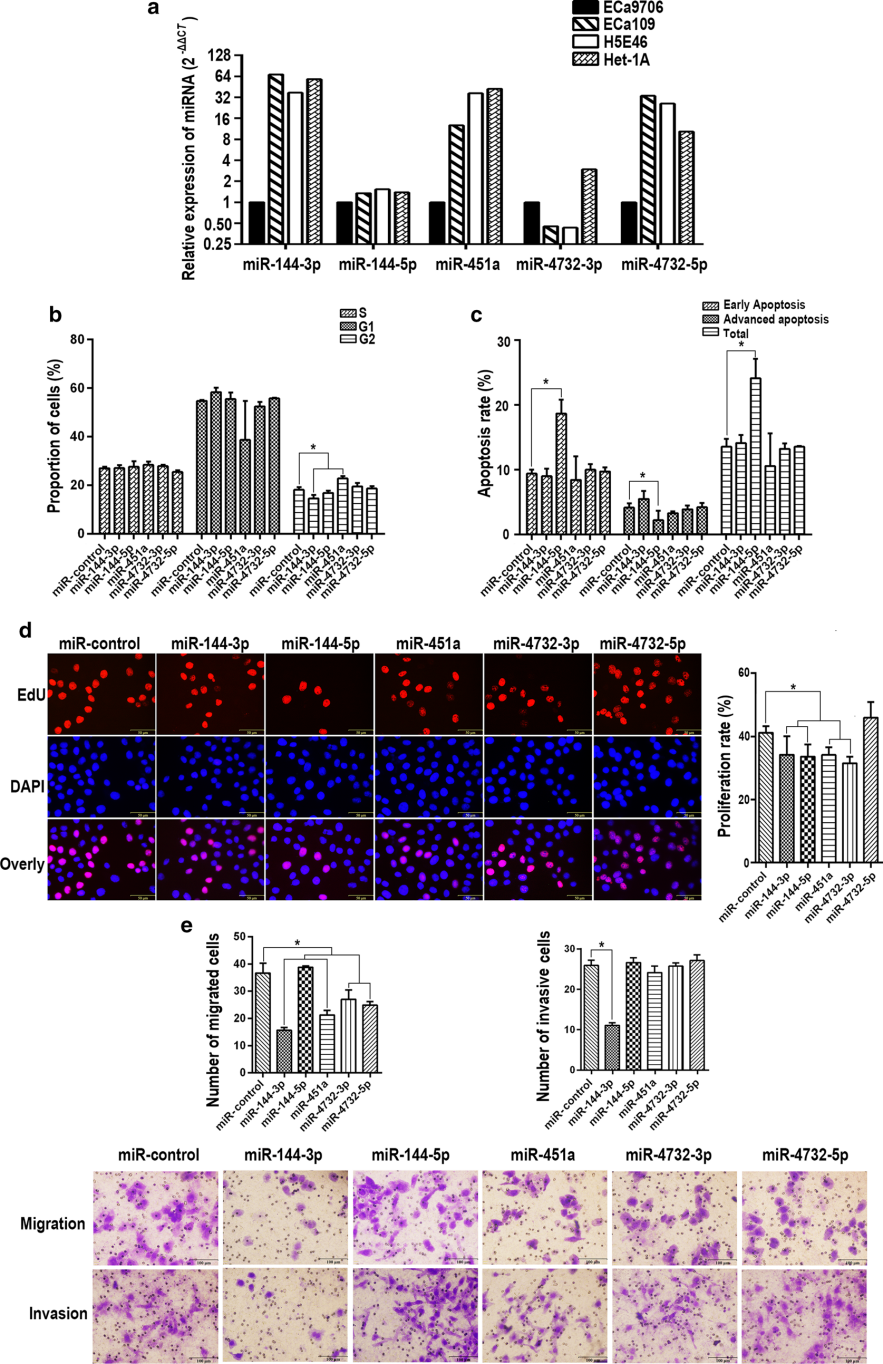
Effects of overexpression of single miRNA on cell functions. **a** Effects of overexpression of sigle miRNA on cell cycle. **b** Effects of overexpression of single miRNA on cell apoptosis. **c** Effects of overexpression of single miRNA on cell proliferation. **d** Effects of overexpression of single miRNA on cell migration and invasion

We detected the expression of miR-144/451 in ECa9706, ECa109, H5E46 and Het-1A. miR-144-3p, miR-144-5p, miR-451a and miR-4732-3p were low expressed (Fig. 1a), Eca9706 were selected for further experiment.

### Transfection efficiency of miRNA mimics

In ECa9706, miRNA mimics significantly up-regulated expression of miR-144-3p, miR-144-5p, miR-451a, miR-4732-3p and miR-4732-5p. Fold changes for all these five miRNAs were more than 1000.

### Effects of individual miRNAs on biological function

miR-144-3p decreased the proportion of cells in G2 phase, but in cells over-expressing miR-451a, the proportion of cells in G2 increased. In cells overexpressing miR-144-3p and miR-451a there are (14.62 ± 1.41)% and (22.84 ± 0.97)% cells in G2 phase respectively, while in control the proportion is (18.20 ± 1.12)% (Fig. 1b). miR-144-3p significantly promoted cell apoptosis with the early apoptosis rate of (18.70 ± 2.11)%, while in control, the early apoptosis rate was (9.00 ± 1.15)% (Fig. 1c). Proliferation rate of cells overexpressing miR-144-3p, miR-144-5p, miR-451a and miR-4732-3p were (34.18 ± 5.83)%, (33.56 ± 3.94)%, (34.15 ± 2.94)% and (31.50 ± 2.01)% respectively, in control the proliferation rate was (41.16 ± 2.13)% (Fig. 1d). For cell migration, compared with the number of migrated cells of 36.67 ± 3.58 in control, miR-144-3p, miR-451a, miR-4732-3p and miR-4732-5p inhibited cell migration, the number of cells passed through the membrane were 15.63 ± 1.00, 21.27 ± 1.70, 26.97 ± 3.47 and 24.87 ± 1.36 respectively. No difference were observed between cells over-expressing miR-144-5p and the control (Fig. 1e). For cell invasion, miR-144-3p obviously inhibited invasive ability, only 12.07 ± 1.10 cells passed through reconstituted basement membrane in cells overexpressing miR-133-3p, while the number was 25.90 ± 2.26 in control. For cell proliferation, miR-144-3p, miR-144-5p, miR-451a and miR-4732-3p significantly inhibited proliferation of ECa9706 (Fig. 1e).

### Expression of miR-144/451 cluster in stable cell line

In cells overexpressing pri-miR-144/451, miR-144-3p, miR-144-5p and miR-451a were significantly up-regulated, the fold change were 152.22, 699.41 and 600.49 respectively. No significant difference of the expression of miR-4732-3p and miR-4732-5p were found between pri-miR-144/451 and the control. miR-4732-3p and miR-4732-5p seems not to be the member of miR-144/451 cluster (Table 2).

**Table 2:** Expression of miR-144/451 in cells over-expressing pri-miR-144/451

miRNA	Δ*C*_*T*_		ΔΔ*C*_*T*_	2^−ΔΔC*T*^	*P* value
	Mimic	Control			
miR-144-3p	19.65 ± 0.46	26.90 ± 0.58	− 7.25 ± 0.46	152.22	< 0.001
miR-144-5p	16.85 ± 0.40	26.30 ± 0.77	− 9.45 ± 0.50	699.41	< 0.001
miR-451a	13.66 ± 0.36	22.89 ± 0.51	− 9.23 ± 0.36	600.49	< 0.001
miR-4732-3p	16.69 ± 0.15	17.13 ± 0.36	− 0.45 ± 0.23	1.37	0.157
miR-4732-5p	19.50 ± 0.46	20.87 ± 0.05	− 1.37 ± 0.27	2.58	0.025
pri-miRNA	10.50 ± 0.14	15.71 ± 1.26	− 5.21 ± 0.73	37.01	0.002

### Effects of miR-144/451 on biological function

**Fig. 2:**
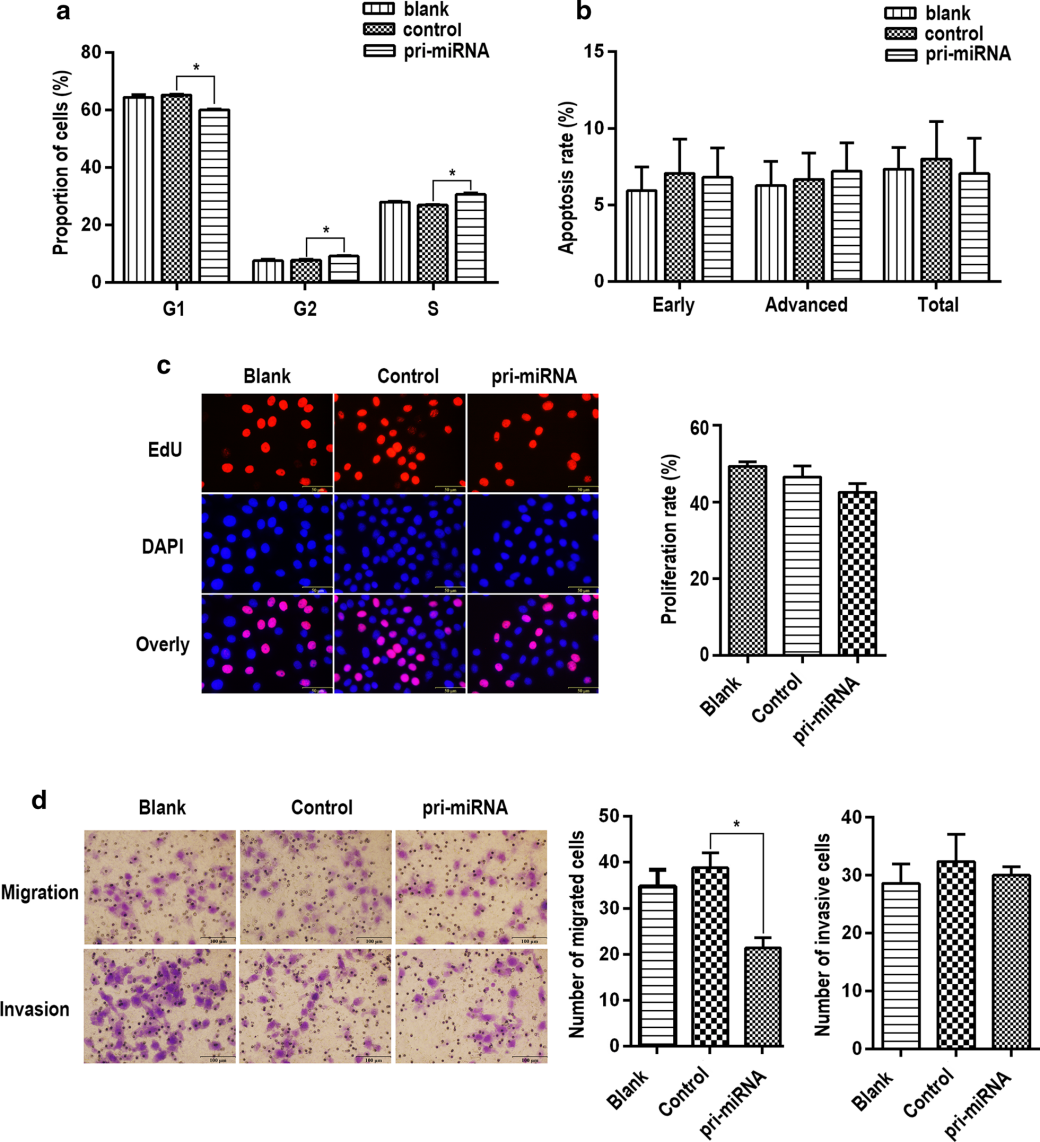
Effects of overexpression of miR-144/451 on cell functions. **a** Effects of overexpression of the cluster on cell cycle. **b** Effects of overexpression of the cluster on cell apoptosis. **c** Effects of overexpression of the cluster on cell proliferation. **d** Effects of overexpression of the cluster on cell migration and invasion

Overexpression of miR-144/451 increased the proportion of cells in phase of G2 and S, thus cells in G1 apparently reduced (Fig. 2a). No difference of apoptosis rate between cell over-expressing miR-144/451 and the control cell were observed (Fig. 2b). Overexpression of miR-144/451 have no effect on cell proliferation and migration (Fig. 2c, d). Transwell assay showed overexpression of miR-144/451 obviously inhibited cell invasion, the inhibition rate reached 50% (Fig. 2d).

### Effects of miR-144/451 on gene expression

**Fig. 3:**
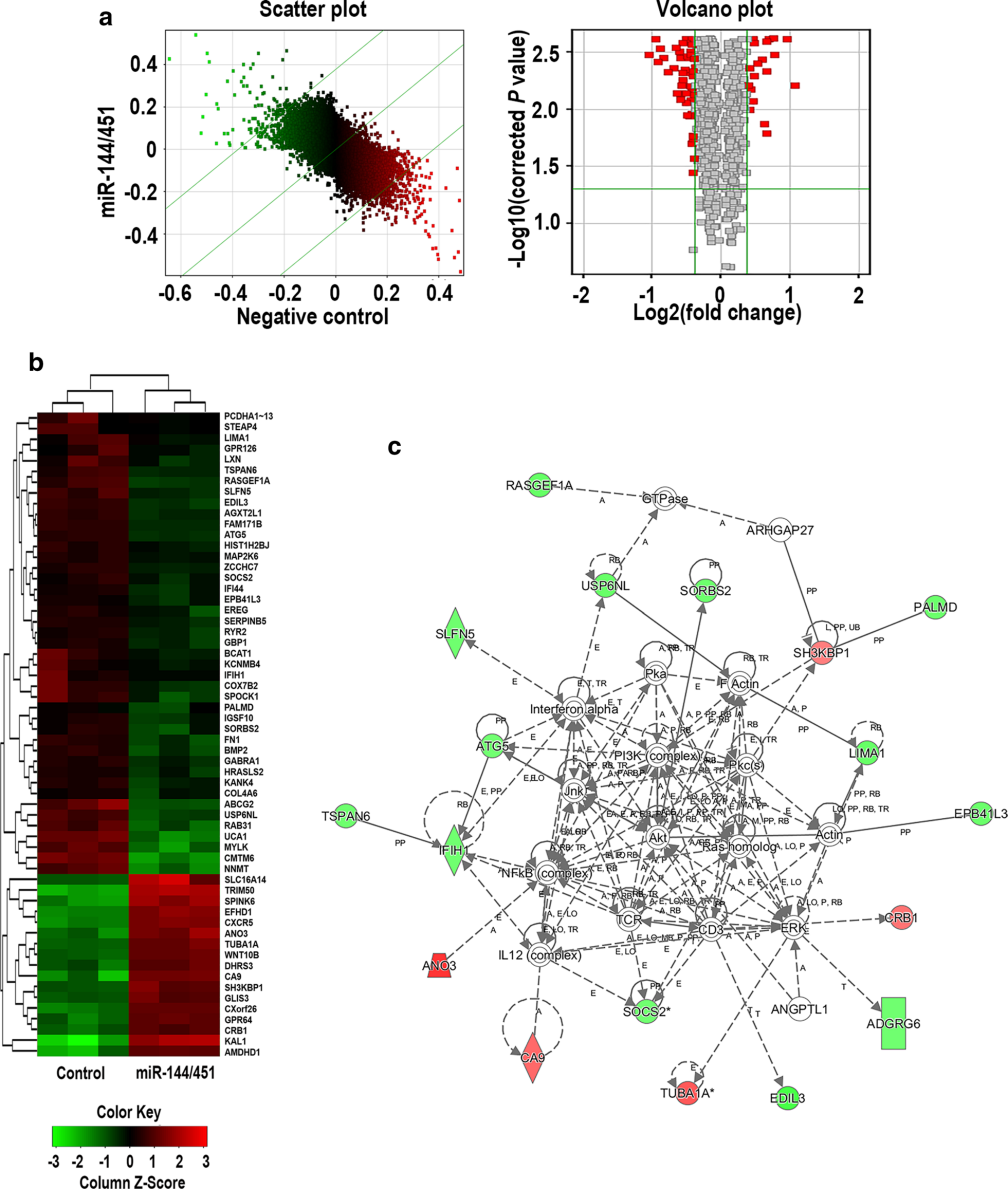
Eeffects of overexpression of miR-144/451 on gene expression. **a** Scatter plot and volcano of differently expressed mRNAs detected by microarray. **b** Heatmap of differently expressed mRNAs. **c** Predicted network regulated by miR-144/451

According to the standard of |Fold change| ≥ 1.3 and *P*-value < 0.05, there are 17 up-regulated and 57 down-regulated genes in cells overexpressing miR-144/451 were detected (Fig. 3a, b), detailed data were shown in Additional file 1: Table S1.

### Possible upstream regulators of the differently expressed mRNAs

IPA were used for analysing the possible upstream regulators, TGFB1, TNF, MAPK1, ERK, TP53, P38 MAPK, SMAD3, TCF/LEF, MMP1, EGFR, MMP2, WNT5A were predicted to be possible upstream regulators, detailed data were shown in Additional file 2: Table S2. According to the predicted interaction of moleculars, we predicted possible networks, the most enriched network were given as Fig. 3c.

### Effect of miR-144-451 gene cluster on key protein

**Fig. 4:**
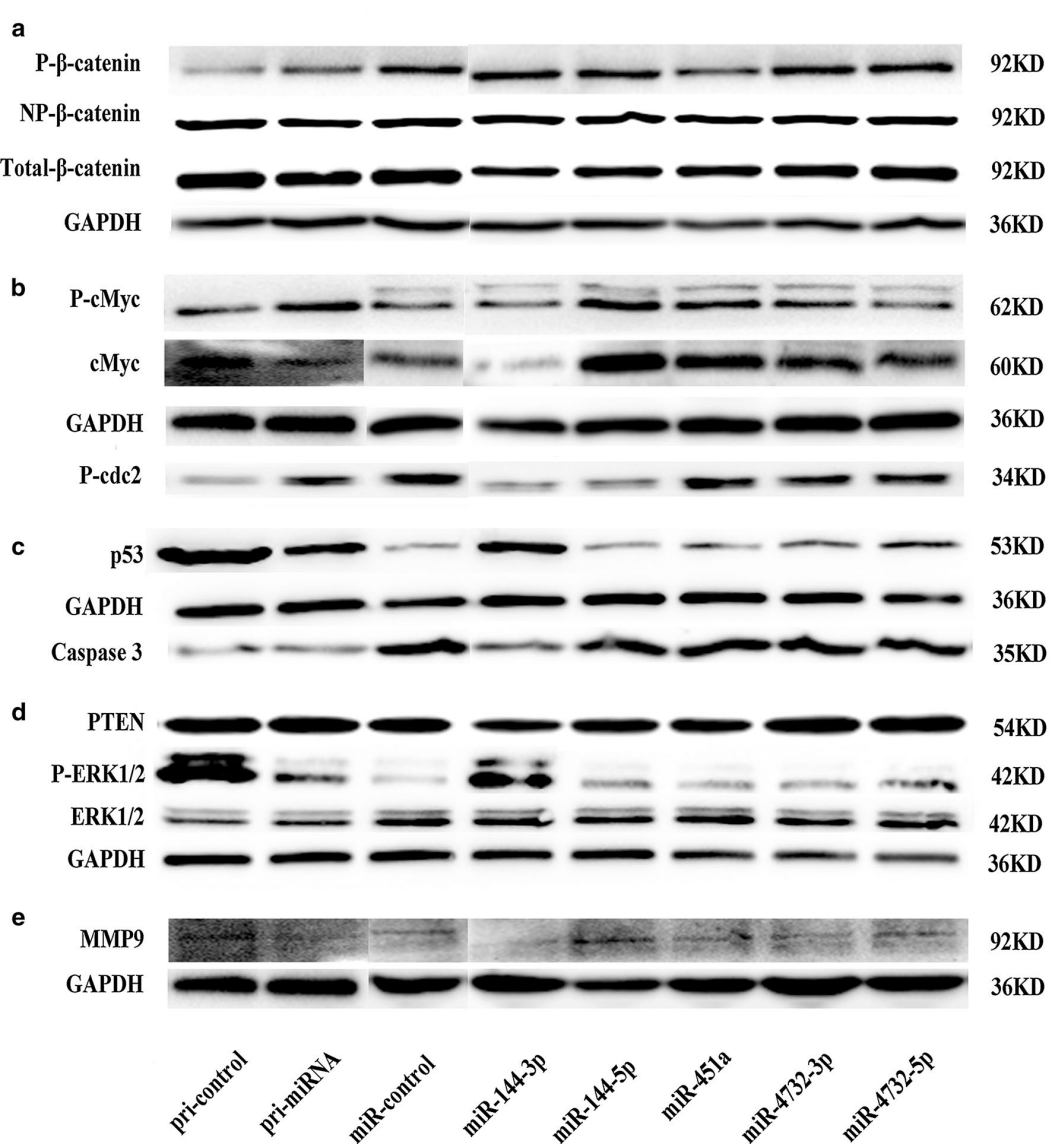
Effect of miR-144/451 cluster onexpression of possible key proteins. **a** Expression of β-catenin. **b** Expression of c-Myc and phosphorylated cdc2. **c** Expression of MAPK/ERK pathway related proteins. **d** Expression of p53 and caspase3. **e** Expression of MMP9

Stable transgenic and miRNA mimic transfected cells were collected individually. Protein expression of selected cells were detected using Western Blot. The expression of protein was evaluated by integrated option density (IOD), Relative expression were evaluated using the ratio of target protein and internal reference protein (Additional file 3: Table S3). Overexpression of miR-144/451 and individual miRNA have no effect on expression of total and unphosphorylated β-catenin. miR-144-3p, miR-144-5p, miR-451a, miR-4732-3p and miR-4732-5p slightly increased the expression of phosphorylated β-catenin with the IOD of 1.01, 1.00, 1.00, 0.99 and 0.99 respectively, while the IOD for control was 0.92. In consistent, in cells over-expressing pri-miR-144/451, the expression of phosphorylated β-catenin was up-regulated (0.73 vs 0.53) (Fig. 4a).

miR-144-3p and miR-451 decreased the expression of total cMyc, but in miR-451a overexpressed cell total cMyc was down-regulated. miR-144-3p decreased the expression of phosphorylated c-Myc, but the cluster increased the expression of phosphorylated c-Myc (Fig. 4b).

Expression of p53 was increased in cells over-expressing miR-4732-5p and pri-miR-144/451, miR-144-3p decreased the expression of non-activated Caspase3 (Fig. 4c).

No difference of expression of PTEN and ERK1/2 were observed among cells overexpressing miRNA and miR-144/451 cluster and the control cells. miR-144-3p, miR-451a, miR-4732-3p and miR-144/451 cluster decreased the expression of phosphorylated ERK1/2 (Fig. 4d).

miR-144-3p, miR-4732-5p and miR-144/451 decreased the expression of MMP9 (Fig. 4e). The expression of phosphorylated cdc2 was decreased in cells over-expressing miR-144-3p and miR-144-5p but increased in cells overexpressing miR-144/451 (Fig. 4b).

## Discussion

miRNA cluster refers to miRNAs encoded closely on chromosome, these miRNAs commonly share the same promoter, and exhibit the similar expression pattern and function of regulating gene expression at the level of posttranscription. It has been reported miR-144-3p and miR-451a played as the tumor suppressor in lung cancer, gastric cancer, colorectal cancer, liver cancer and many other cancers, miR-144-5p has been reported to be tumor suppressor in bladder cancer [11–22]. In consistent, in present study, we discovered miR-144-3p, miR-144-5p, miR-451a played a tumor suppressor role in esophageal cancer.

Considering the complexity the regulation of moleculars, the function of individual miRNA may not exactly reflect real function of the cluster. The maturity of miRNA needs undergo pri-miRNA transcripts, pre-miRNA and mature miRNA in turn [23], so we established cells over-expressing miR-144/451 by over-expressing pri-miR-144/451. In cells overexpressing pri-miR-144/451, the expression of miR-4732-3p and miR-4732-5p almost unchanged, considering the biological function of this two miRNAs are not remarkable as the other three, we infered that miR-4732-3p and miR-4732-5p were not the real member of miR-144/451 cluster.

The function of miR-144/451 cluster were not the summation of the individual miRNA, which were not entirely consistent with the individual miRNA. miR-144-3p, miR-451a, miR-4732-3p and miR-4732-5p inhibited cell migration, miR-144-3p, miR-144-5p, miR-451a and miR-4732-3p inhibited cell proliferation, however, no change of cell migration and proliferation were observed in cells over-expressing miR-144/451. The fold change in mimic transfected cell is larger than that in stable transfected cell, the complex interaction among these miRNAs may be responsible for this result.

To better understand the possible regulatory mechanism of miR-144/451, genome expression array were performed to detect the changed mRNA profiles between cells overexpressing miR-144/451 and control. The fold change of expression of mRNAs was not large in general, biological analysis suggested miR-144/451 is closely related with phosphorylation, miR-144/451 seemed mainly regulated post transcription. According to the result and the result of cell function, we selected the corresponding key proteins for verification.

Wnt signaling pathway regulates cell differentiation, proliferation, migration and many other functions, some components of Wnt signaling pathway were also implicated in other signaling pathways [24–26]. As a key component of Wnt signaling pathway, β-catenin regulates the activation of TCF/LEF, which plays important roles in transcription regulation [27]. Phosphorylated β-catenin was unstable and could be degraded by 26S protease, non-phosphorylated β-catenin was not easy to be degraded which leads to the accumulation of β-catenin, thereby phosphorylation state regulates transcription regulation. Phosphorylation of Thr41, Ser37 and Ser33 is the main degradation pathway [28, 29]. In present study, miR-144-3p, miR-144-5p and miR-451a increased the expression of phosphorylated β-catenin, these miRNAs played a synergistic effect on the inhibition of Wnt/β-catenin signaling pathway.

As transcription factor, c-Myc was reported to inhibit cell apoptosis and promote cell proliferation by activating key protein implicated in apoptosis and proliferation, such as RAS, RAF, BCL-2 and c-ABL [29–34]. Phosphorylation of the Ser62 Strengthened stability of c-Myc, therefore prolonged the half-life of c-Myc [35]. In this study, miR-144-3p decreased the expression of c-Myc, miR-144-5p and miR-451a increased the expression of c-Myc, miR-144/451 decreased the expression of c-Myc, however the expression of phosphorylated c-Myc was down-regulated, suggesting that the low expression of c-Myc may be mainly due to the inhibition of transcriptional translation but not degradation.

MAPK/ERK signaling pathway played important roles in a variety of tumors, by regulating cyclinD1, ERK regulates the transformation of cell cycle from G1 to S phase. The signaling pathway is reported to be closely related with matrix metalloproteinase (MMPs) which is essential for cell invasion [36, 37]. As the upstream regulatory protein of MAPK/ERK signaling pathway, in this study miR-144/451 have no effect on expression of PTEN [38], while the expression of phosphorylated ERK1/2 was decreased by over-expressing of miR-144/451 cluster, no change of the expression of Non- phosphorylated ERK1/2 were observed. The inhibition of MAPK/ERK signaling pathway may mainly caused by posttranscriptional modification of ERK1/2, miR-144-3p and miR-451a played a synergistic role in the inhibition of MAPK/ERK signaling pathway.

cdc2 plays important roles on regulating cell cycle. In advanced G2 phase, cdc2 combined with cyclinB to promote cell entry into M phase [39, 40]. Dephosphorylation of Tyr15 is essential to the activation of cdc2. miR-144/451 increased the expression of p-cdc2, which was consistent with the change of cell cycle.

In this study, over-expression of miR-144/451 up-regulated the expression of p53, interestingly, in miR-144-3p, miR-144-5p and miR-451a over-expressing cells no up-regulation of p53 were observed. miR-144/451 cluster is not a simple functional superposition of individual miRNAs. As key protein regulating cell apoptosis, Caspase3 exists in normal cells in the form of inactive zymogen, at early stage of apoptosis, Caspase3 can be activated and split into 12KD and 17KD products [41, 42]. In this study, miR-144-3p decreased the expression of 34KD Caspase3, no change in the expression of Caspase3 were observed in stable cell over-expressing miR-144/451, which were consistent with results of cell apoptosis.

## Conclusion

miR-144-451 inhibited invasion and cell cycle of EC9706. miR-144-3p prometed the apoptosis of EC9706, miR-451a can lead to the arrest of G2 phase, miR-144-3, miR-451a,miR-4732-3p inhibited the migration of cell, miR-144-3p inhibited the invasion of cell, the proliferation can be inhibited by miR-144-3p, miR-144-5p, miR-451a and miR-4732-3p. Wnt and MAPK/ERK/c-Myc signaling pathways were inhibited by miR-144-451 cluster, at while, miR-144/451 cluster downregulated the expression of MMP9 and upregulated the expression of p-cdc2 which may participated in the process of cell cycle arrest and invasion inhibition.

## Additional files

**Additional file 1: Table S1.** Abnormally expressed mRNAs in miR-144/451 overexpressing.

**Additional file 2: Table S2.** Upstream regulators of mRNAs abnormally expressed.

**Additional file 3: Table S3.** Relative expression of proteins.

## Abbreviations

EdU: 5-ethynyl-2′-deoxyuridine
IPA: Ingenuity Pathway Analysis
BCA: bicinchoninic acid
MMLV: Moloney Murine Leukemia Virus
IOD: integrated option density
