# Supplementary material for: miR-144/451 cluster plays an oncogenic role in esophageal cancer by inhibiting cell invasion
Source: Cancer Cell Int. 2018 Nov 15;18:184. doi: 10.1186/s12935-018-0679-8 (PMC6238332; doi:10.1186/s12935-018-0679-8)
Supplement: Supplementary file 1 — Additional file 1: Table S1. Abnormally expressed mRNAs in miR-144/451 overexpressing. [file 12935_2018_679_MOESM1_ESM.docx]

Table S1 Abnormally expressed mRNAs in miR-144/451 overexpressing cells

| Gene Symbol | Fold change | Regulation | Corrected *P*-value |
| --- | --- | --- | --- |
| ANO3 | 2.1075914 | up | 0.00615153 |
| SPINK6 | 1.9382592 | up | 0.002423478 |
| TUBA1A | 1.7065046 | up | 0.003334417 |
| GPR64 | 1.6107396 | up | 0.002423478 |
| KAL1 | 1.5810243 | up | 0.002544856 |
| CXorf26 | 1.5697106 | up | 0.004620182 |
| AMDHD1 | 1.5695348 | up | 0.016141133 |
| CA9 | 1.5476222 | up | 0.01332341 |
| SLC16A14 | 1.5042344 | up | 0.003624963 |
| CRB1 | 1.4334923 | up | 0.003930402 |
| WNT10B | 1.3965577 | up | 0.002605445 |
| GLIS3 | 1.374969 | up | 0.008430082 |
| DHRS3 | 1.3704695 | up | 0.002423478 |
| EFHD1 | 1.3398459 | up | 0.006710441 |
| TRIM50 | 1.3259677 | up | 0.004331487 |
| SH3KBP1 | 1.3232164 | up | 0.010011354 |
| CXCR5 | 1.3111075 | up | 0.006114862 |
| ANO3 | 2.1075914 | up | 0.00615153 |
| SPINK6 | 1.9382592 | up | 0.002423478 |
| TUBA1A | 1.7065046 | up | 0.003334417 |
| GPR64 | 1.6107396 | up | 0.002423478 |
| KAL1 | 1.5810243 | up | 0.002544856 |
| CXorf26 | 1.5697106 | up | 0.004620182 |
| AMDHD1 | 1.5695348 | up | 0.016141133 |
| SERPINB5 | 2.0823283 | down | 0.00331294 |
| SPOCK1 | 1.9426762 | down | 0.002423478 |
| KANK4 | 1.8864775 | down | 0.003803655 |
| FN1 | 1.854098 | down | 0.00293006 |
| COL4A6 | 1.7799174 | down | 0.003540285 |
| EDIL3 | 1.7099894 | down | 0.00471377 |
| CMTM6 | 1.614755 | down | 0.004331487 |
| EREG | 1.5526112 | down | 0.007230576 |
| ABCG2 | 1.5194082 | down | 0.003090064 |
| UCA1 | 1.5000906 | down | 0.008140425 |
| KCNMB4 | 1.4807876 | down | 0.005550714 |
| COX7B2 | 1.4685093 | down | 0.002605445 |
| RYR2 | 1.4660435 | down | 0.004797031 |
| AGXT2L1 | 1.4458947 | down | 0.004495896 |
| LXN | 1.427332 | down | 0.003540285 |
| HRASLS2 | 1.4247745 | down | 0.007270818 |
| RASGEF1A | 1.4212955 | down | 0.002605445 |
| BCAT1 | 1.4201179 | down | 0.008735759 |
| LIMA1 | 1.4168521 | down | 0.002423478 |
| IGSF10 | 1.4118012 | down | 0.00342732 |
| USP6NL | 1.409242 | down | 0.01122572 |
| EPB41L3 | 1.3818742 | down | 0.011471971 |
| IFI44 | 1.3678229 | down | 0.004769153 |
| PALMD | 1.3656564 | down | 0.008380215 |
| SORBS2 | 1.3605615 | down | 0.003090064 |
| GABRA1 | 1.3558168 | down | 0.006229249 |
| NNMT | 1.3534051 | down | 0.003334417 |
| PCDHA1~13 | 1.3509469 | down | 0.006839956 |
| STEAP4 | 1.3488837 | down | 0.002715352 |
| TSPAN6 | 1.3466108 | down | 0.002423478 |
| RAB31 | 1.3441049 | down | 0.010988258 |
| SLFN5 | 1.3428869 | down | 0.020008478 |
| GBP1 | 1.3306893 | down | 0.004620182 |
| GPR126 | 1.3301389 | down | 0.005116347 |
| FAM171B | 1.3299423 | down | 0.017205706 |
| MYLK | 1.3286389 | down | 0.03598922 |
| IFIH1 | 1.3270254 | down | 0.016896976 |
| HIST1H2BJ | 1.3249687 | down | 0.018158425 |
| SOCS2 | 1.3249341 | down | 0.018261353 |
| MAP2K6 | 1.3219355 | down | 0.004732693 |
| ZCCHC7 | 1.3211436 | down | 0.027003072 |
| ATG5 | 1.3086975 | down | 0.00358425 |
| BMP2 | 1.3016067 | down | 0.004299854 |
