# Supplementary material for: miR-144/451 cluster plays an oncogenic role in esophageal cancer by inhibiting cell invasion
Source: Cancer Cell Int. 2018 Nov 15;18:184. doi: 10.1186/s12935-018-0679-8 (PMC6238332; doi:10.1186/s12935-018-0679-8)
Supplement: Supplementary file 2 — Additional file 2: Table S2. Upstream regulators of mRNAs abnormally expressed. [file 12935_2018_679_MOESM2_ESM.docx]

Table S2 Upstream Regulators of mRNAs abnormally expressed miR-144/451

| Upstream regulator | Molecule type | P-value |
| --- | --- | --- |
| TGFB1 | growth factor | 0.000000112 |
| TNF | cytokine | 0.000000703 |
| MAPK1 | kinase | 0.000028 |
| ERBB2 | kinase | 0.0000425 |
| WWTR1 | transcription regulator | 0.0000673 |
| IRF3 | transcription regulator | 0.0000838 |
| EPAS1 | transcription regulator | 0.000145 |
| beta-estradiol | chemical - endogenous mammalian | 0.000151 |
| ERK | group | 0.000338 |
| PKD1 | ion channel | 0.00113 |
| MAP3K3 | kinase | 0.00182 |
| TP53 | transcription regulator | 0.00327 |
| MMP2 | peptidase | 0.00407 |
| BDNF | growth factor | 0.00782 |
| P38 MAPK | group | 0.0138 |
| SMAD3 | transcription regulator | 0.0201 |
| TCF/LEF | group | 0.0209 |
| ERBB4 | kinase | 0.0217 |
| VEGFA | growth factor | 0.0281 |
| WNT5A | cytokine | 0.0314 |
| PRKAA2 | kinase | 0.0336 |
| ZNF148 | transcription regulator | 0.0337 |
| Collagen type II | complex | 0.0363 |
| HDAC4 | transcription regulator | 0.0365 |
| NDN | transcription regulator | 0.0388 |
| TGFB1I1 | transcription regulator | 0.0414 |
| SMAD7 | transcription regulator | 0.0418 |
| MMP1 | peptidase | 0.0439 |
| EGFR | kinase | 0.0459 |
