# Supplementary material for: miR-144/451 cluster plays an oncogenic role in esophageal cancer by inhibiting cell invasion
Source: Cancer Cell Int. 2018 Nov 15;18:184. doi: 10.1186/s12935-018-0679-8 (PMC6238332; doi:10.1186/s12935-018-0679-8)
Supplement: Supplementary file 3 — Additional file 3: Table S3. Relative expression of proteins. [file 12935_2018_679_MOESM3_ESM.docx]

Table S3 Relative expression of proteins

| Protein | Pri-miRNA | pri-control | miR-control | miR-144-3p | miR-144-5p | miR-451a | miR-4732-3p | miR-4732-5p |
| --- | --- | --- | --- | --- | --- | --- | --- | --- |
| P-β-catenin | 0.73 | 0.53 | 0.92 | 1.01 | 1.00 | 1.00 | 0.99 | 0.99 |
| NP-β-catenin | 2.03 | 2.01 | 1.95 | 1.97 | 2.01 | 1.99 | 1.98 | 1.99 |
| Total-β-catenin | 1.47 | 1.53 | 1.56 | 1.49 | 1.48 | 1.44 | 1.49 | 1.50 |
| P-cMyc | 0.79 | 0.26 | 0.51 | 0.39 | 1.23 | 1.09 | 0.85 | 0.73 |
| cMyc | 0.72 | 1.11 | 0.82 | 0.35 | 1.03 | 0.91 | 0.87 | 0.75 |
| P-cdc2 | 0.62 | 0.18 | 0.94 | 0.50 | 0.56 | 0.93 | 0.86 | 0.88 |
| PTEN | 1.53 | 1.56 | 1.49 | 1.47 | 1.50 | 1.50 | 1.48 | 1.43 |
| P-ERK1/2 | 0.70 | 1.03 | 0.86 | 0.53 | 0.82 | 0.38 | 0.37 | 0.98 |
| ERK1/2 | 0.61 | 0.63 | 0.65 | 0.67 | 0.64 | 0.67 | 0.62 | 0.59 |
| p53 | 0.82 | 0.63 | 0.72 | 0.66 | 0.64 | 0.71 | 0.75 | 0.87 |
| Caspase3 | 0.23 | 0.25 | 1.02 | 0.70 | 0.98 | 0.97 | 0.96 | 0.99 |
| MMP9 | 0.16 | 0.25 | 0.10 | 0.27 | 0.30 | 0.15 | 0.31 | 0.28 |
